# Reproducibility of shear wave elastography (SWE) in patients with chronic liver disease

**DOI:** 10.1371/journal.pone.0185391

**Published:** 2017-10-12

**Authors:** Marcello Mancini, Angelo Salomone Megna, Monica Ragucci, Massimo De Luca, Giuseppina Marino Marsilia, Gerardo Nardone, Pietro Coccoli, Anna Prinster, Lorenzo Mannelli, Emilia Vergara, Serena Monti, Raffaele Liuzzi, Mariarosaria Incoronato

**Affiliations:** 1 Institute of Biostructure and Bioimaging, National Research Council, Naples, Italy; 2 Division of Infectious Diseases, Rummo Hospital, Benevento, Italy; 3 Liver Unit, AORN Cardarelli, Naples, Italy; 4 Pathology Unit, Cardarelli Hospital, Napoli, Italy; 5 Department of Clinical Medicine and Surgery, Gastroenterology Unit, Federico II University, Naples, Italy; 6 Department of Radiology, Memorial Sloan-Kettering Cancer Center, New York, United States of America; 7 Istituto di Ricovero e Cura a Carattere Scientifico SDN (IRCCS SDN), Naples, Italy; 8 Dipartimento Assistenziale Integrato di Diagnostica morfologica e funzionale, Radioterapia, Medicina Legale, A.O.U. Federico II, Naples, Italy; Universita degli Studi di Pisa, ITALY

## Abstract

The presence of significant fibrosis is an indicator for liver disease staging and prognosis.

The aim of the study was to determine reproducibility of real-time shear wave elastography using a hepatic biopsy as the reference standard to identify patients with chronic liver disease. Forty patients with chronic liver disease and 12 normal subjects received shear wave elastography performed by skilled operators. Interoperator reproducibility was studied in 29 patients. Fibrosis was evaluated using the Metavir score. The median and range shear wave elastography values in chronic liver disease subjects were 6.15 kPa and 3.14–16.7 kPa and were 4.49 kPa and 2.92–7.32 kPa in normal subjects, respectively. With respect to fibrosis detected by liver biopsy, shear wave elastography did not change significantly between F0 and F1 (p = 0.334), F1 and F2 (p = 0.611), or F3 and F4 (0.327); a significant difference was observed between the F0-F2 and F3-F4 groups (p = 0.002). SWE also correlated with inflammatory activity (Rs = 0.443, p = 0.0023) and ALT levels (Rs = 0.287, p = 0.0804). Age, sex and body mass index did not affect shear wave elastography measurements. Using receiver operator characteristic curves, two threshold values for shear wave elastography were identified: 5.62 kPa for patients with fibrosis (≥F2; sensitivity 80%, specificity 69.4%, and accuracy 77%) and 7.04 kPa for patients with severe fibrosis (≥F3; sensitivity 88.9%, specificity 81%, and accuracy 89%). Overall interobserver agreement was excellent and was analysed using an interclass correlation coefficient (0.94; CI 0.87–0.97).This study shows that shear wave elastography executed by skilled operators can be performed on almost all chronic liver disease patients with high reproducibility. It is not influenced by age, sex or body mass index, identifies severely fibrotic patients and is also related to inflammatory activity.

## Introduction

Chronic liver diseases (CLDs) are an important public health issue. The prevalence of CLD in Europe was estimated to be as high as 5.82% and that of cirrhosis is 0.1%, corresponding to 14–26 new cases per 100,000 inhabitants per year and an estimated 170,000 deaths per year [1]. Liver disease-associated mortality in Europe is comparable with that of other diseases considered to be major public health concerns, such as breast cancer, colon and rectum cancers and chronic obstructive pulmonary disease [2–4].

The presence of significant fibrosis is a hallmark for liver disease staging and prognosis [5]. Currently, CLD treatment requires a correct assessment of liver fibrosis [6,7]. Indeed, fibrosis progression occurs within three years in 33% of untreated hepatitis C patients, even in patients with persistently normal alanine aminotransferase [8]. Interestingly, new antiviral treatments that generate a sustained viral response can induce liver fibrosis regression [9–11]. Therefore, fibrosis evaluation is a critical point when doctors must also consider that patients with mild disease might be eligible for antiviral therapy in the near future.

Currently, the reference standard for assessing patients is a liver biopsy, which is considered the most specific test to evaluate the nature and severity of CLD and is useful to monitor treatment efficacy [8]. The biopsy specimen size varies from 1 and 3 cm in length and between 1–2 mm in diameter; therefore, the sample represents 1/50,000 of the total liver mass [8]. However, the biopsy is an invasive procedure that is possibly fraught with secondary effects, and approximately 1 to 3% of patients require hospitalization for complications. The mortality rate among patients after percutaneous liver biopsy is approximately 1 in 10,000 to 1 in 20,000; thus it cannot be repeated frequently to monitor liver disease [12, 13].

Moreover, biopsy sampling error and sample size are major sources of bias during liver fibrosis assessment [14]. Therefore, there is increasing interest in developing new, non-invasive methods to evaluate CLD patients as an alternative to biopsy, with a focus on the elastographic methods.

Ultrasound (US)-based elastography samples a liver volume 150 times larger than a biopsy specimen. Shear wave elastography (SWE) uses the measurement of acoustically generated shear wave propagation speeds in the tissue to estimate liver stiffness with the advantage of simultaneous anatomic B-Mode US imaging [15]. This imaging technology mechanically excites using short-duration acoustic pulses in a region of interest (ROI) chosen by the examiner. This produces shear waves that spread out from the region of production perpendicular to the acoustic push pulse and is generated by localized, micron-scale displacements in the tissue [15–19].

Simultaneously, detection waves of a lower intensity than the push pulse (1:100) are generated. The push pulse uses a few hundred cycles and different voltages compared to the short cycle B-mode pulse. The moment of interaction between the shear waves and the detection waves marks the time period elapsed between shear wave generation until they cross the entire ROI. By recording the shear wave front at several locations and correlating these measurements with the elapsed time, the shear wave velocity (SWV) (m/s) can be quantified; generally, the stiffer a tissue region, the greater the SWV as it travels through this region. Thus, the measured SWV is an intrinsic and reproducible tissue property.

The aim of the study was to determine normal reference values and the reproducibility of real-time SWE using a hepatic biopsy as the gold standard to identify patients with CLD. Moreover, the accuracy of SWE for detecting various fibrosis stages will be assessed.

## Materials and methods

### Patients

This prospective study included 40 chronic patients evaluated in our outpatient clinic between January 2014 and March 2015 who were scheduled for percutaneous liver biopsy and 12 normal subjects.

The exclusion criteria were other active infectious diseases or pregnancy.

Five patients (12.8%) received a diagnosis of liver cirrhosis, 11 patients (28.1%) were diagnosed with viral chronic hepatitis, 7 (17.9%) had alcoholic liver disease, and 17 patients (41.2%) exhibited unexplained abnormalities on their liver test results. None of the normal subjects had a history of liver disease and had normal test results; all of them had a normal abdominal ultrasonography.

All of the subjects received a general abdomen US examination and SWE examination.

All of the patients with suspected chronic hepatic disease were scheduled for a liver biopsy to stage and grade their condition within 6 months before or after the US examination.

The study was approved by the local Ethical Committee of the faculty of Medicine—University of Naples Federico II. The patients provided written informed consent before the beginning of the study.

### Shear wave elastography examination

SWE was performed using the iU22 system (Philips) with a convex broadband probe (C5-1). The elastography of the system (Shear Wave Point Quantification) generates shear waves inside the liver using the acoustic force of a focused US beam. The patient was lying in a lateral decubitus position with the right arm extended above the head for access to the right hypochondrium and to increase the intercostal acoustic window. The probe was placed parallel to the intercostal space with sufficient gel to minimize rib shadowing. An ROI with a box size of 2.0x1.0 cm was positioned within the liver parenchyma under visual control in two-dimensional B-mode at a depth of at least 2 cm below the liver capsule in segments 7 or 8 of the liver, taking care to not include large vasculature or biliary structures. The sub-capsular regions that usually contain larger fibrotic content were avoided. During scanning between ribs, no pressure was applied to the liver, and the patient was asked to stop breathing for a few seconds to minimize motion artefacts. Liver stiffness measurements were performed on the same area of the liver parenchyma. The equipment listed the SWV (m/s) in the ROI as well as the depth at which the measurement was performed. To compute tissue stiffness in kilopascals (kPa), the shear wave velocity (v) was converted into the Shear Modulus G = τ/γ, in which τ is the shear stress and γ is the shear strain based on the relationship G = ρv^2^, in which ρ is the density of the tissue (liver is approximately kg/m^3^). Fifteen measurements were collected at the same location, and a report was generated when a success rate of at least 80% was obtained [20, 21].

The average and median of these measurements expressed in kPa were then used to estimate the degree of liver stiffness for each subject and were correlated with the biopsy Metavir score.

To study interoperator reproducibility in a subgroup of 29 patients, the procedures were performed in the same week by two operators (MM, ASM). The operators were blinded to the results of previous measurements and to biological and histological data.

### Histopathological analysis

A liver specimen was collected with the BIOMOL-16 G, a soft tissue biopsy semiautomatic needle (HS SpA, Aprilia-Italy), using the modified Menghini needle technique. The liver biopsy specimens were fixed in formalin and embedded in paraffin. A specialized pathologist with more than 20 years of experience who was blinded to the SWE values and clinical information reviewed the biopsy specimens. Only biopsy specimens 2 cm long and with a minimum of 11 intact portal tracts were eligible for evaluation [22]. Liver fibrosis was evaluated semi-quantitatively according to the Metavir scoring system [23]. Liver fibrosis was staged using a five-point ordinal scale ranging from 0 to 4 as follows: F0 –no fibrosis; F1 –portal fibrosis without septa; F2—portal fibrosis with few septa but intact architecture; F3 –numerous septa with architecture distortion without cirrhosis; F4 –cirrhosis.

Histological grading of portal inflammation was assessed by Ishak score (0 = no portal inflammation; 1 = mild in some or all portal areas; 2 = moderate in some or all portal areas; 3 = moderate to marked, all portal areas; 4 = marked in all portal areas) [24]. Moreover ALT levels were used as index of hepatic inflammation.

### Statistical analysis

Continuous variables were expressed as the median and range. An unpaired non-parametric Mann-Whitney U test was used to compare data from different groups. Categorical variables were expressed as percentages. The SWE distribution for each class of histopathological parameters analysed was studied using “box plot” diagrams. The Spearman’s rank correlation coefficient (Rs) was used to verify the association of variables.

A receiver operating characteristic (ROC) curve was calculated. The area under the curve (AUC) was used to evaluate test accuracy, and the discrimination value was determined by Youden’s J statistic. Sensitivity, specificity, and positive and negative predictive values were computed using this threshold.

Interobserver agreement of liver elasticity was evaluated in terms of the intraclass correlation coefficient (ICC). Agreement was classified as poor (ICC = 0.00 to 0.40), fair to good (ICC = 0.40 to 0.75) or excellent (ICC>0.75) [24].

For descriptive purposes, according to the method described by Bland and Altman [25], interobserver variability was assessed by plotting the difference between the measurements of the two operators against their means. The 95% confidence intervals (CI) for the differences indicated interobserver variability. All of the statistical tests were two-sided, and a p-value of 0.05 or less was considered statistically significant. The statistical analysis was performed using MedCalc software version 12.7 (MedCalc Software Bvba, NL).

## Results

We analysed data from 40 patients with CLD and 12 normal subjects without a history of liver disease. The demographic, clinical, and biochemical characteristics of the patients and normal subjects are summarized in **Table 1**. Valid SWE measurements were obtained from all but one patient (measurement failed because of ascites). The SWE measurement value in CLD was 6.15 kPa (range 3.14–16.7 kPa), whereas the median liver elasticity for normal subjects was 4.49 kPa (range 2.92–7.32).

**Table 1:** Main clinical and demographic characteristics of patients and healthy subjects who received shear wave elastography and biopsy.

Characteristic	Patients (39)	Healthy subjects (12)
Male gender	22 (56%)	3 (25%)
Age (years)	48.5 (20.0–78.0)	27.5 (25.0–48.0)
Weight (kg)	72.0 (51.0–130.0)	58.0 (52.0–70.0)
Body Mass Index (kg/m^2^)	25.6 (17.9–38.4)	22.2 (18.8–25.0)
Abdominal circumference (cm)	98.0 (77.0–120.0)	89.0 (79.0–93.0)
AST (U/l)	37.5 (14.0–377.0)	15.5 (14.0–17.0)
ALT (U/l)	51.5 (9.0–577.0)	11.0 (9.0–13.0)
GGT(U/L)	81.0 (14.0–856.0)	12.5 (10.0–15.0)
Cholesterol (mg/dl)	180.0 (62.0–342.0)	151.5 (133.0–170.0)
Triglycerides (mg/dl)	120.0 (33.0–353.0)	37.5 (27.0–48.0)
**Aetiology of liver disease**		
HCV	4 (10,2%)	
HBV	7 (17,9%)	
Alcohol-related	7 (17,9%)	
NASH	2 (5,1%)	
Others	19 (48,7%)	
**Shear Wave Elastography**		
Median value (range)	6.15 (3.14–16.66)	4.49 (2.92–7.32)

Of the 39 patients, 5 were assigned Metavir stage F0 (12.8%), 19 were F1 (48.7%), 6 were F2 (15.4%), 4 were F3 (10.2%), and 5 were F4 (12.8%). No significant difference was observed between the liver elasticity of normal subjects and that of F0 patients (median 4.49 kPa versus 5.52 kPa; p = 0.073). Based on this result, we further grouped subjects into only one group of control subjects classified as F0.

The average size of the liver biopsy specimen was 1.2 mm with an average number of 11 portal spaces.

**Fig 1:**
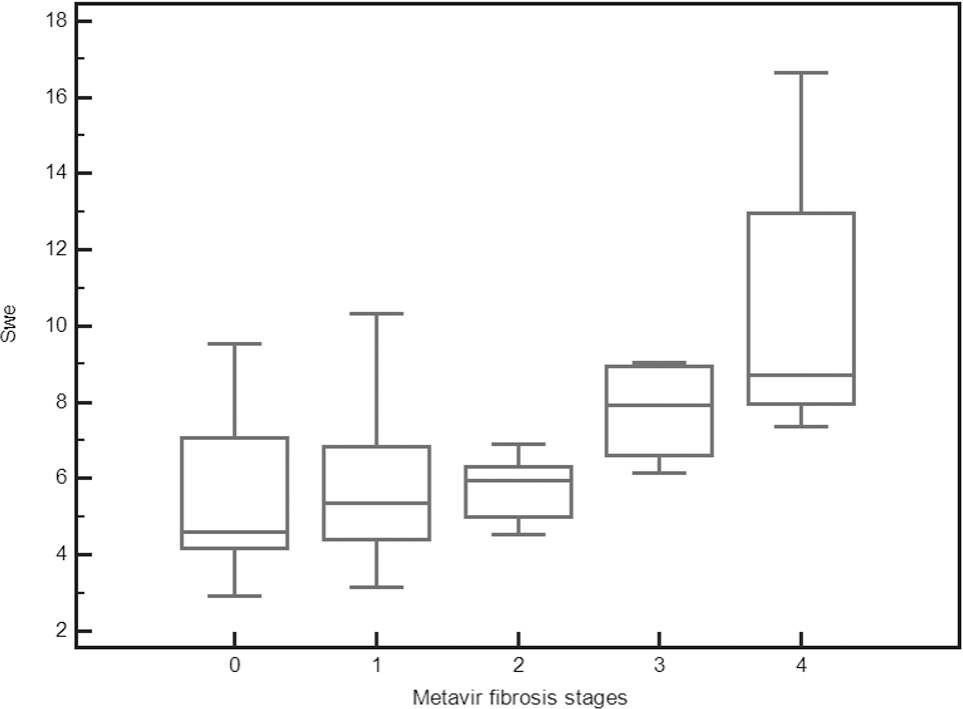
Box-and-whisker plots of shear wave elastography values for each Metavir stage. Liver stiffness values are reported on the y-axis, and Metavir grades are reported on the x-axis. The line through each box represents the median, and the central box represents values from the lower to upper quartiles (25th- 75th percentile). Error bars show minimum and maximum non-extreme values.

**Fig 2:**
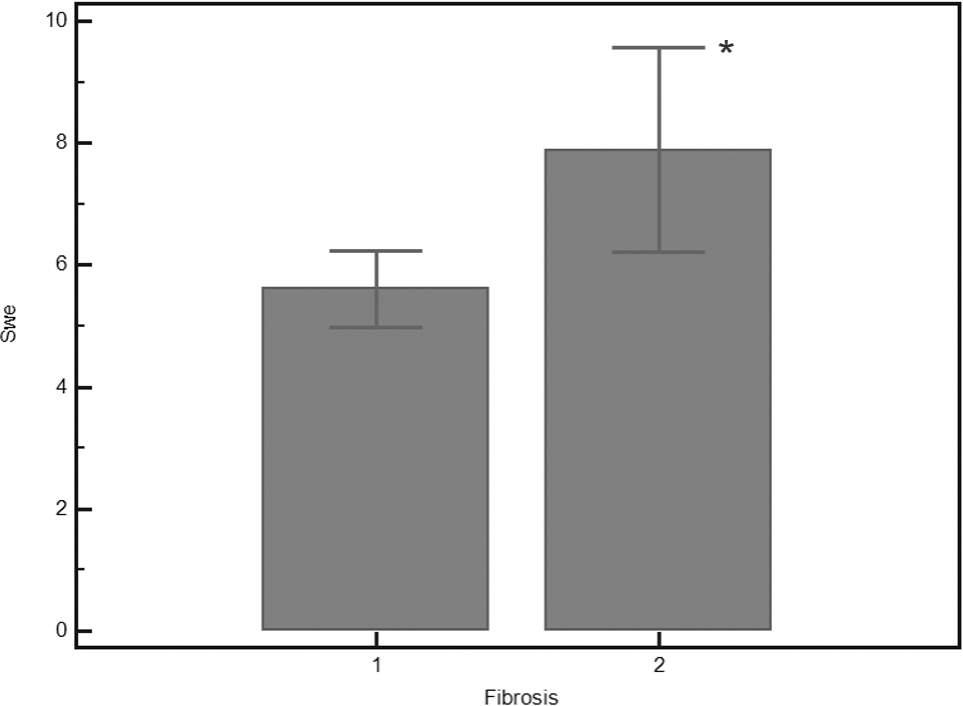
Comparison of shear wave elastography in 36 patients classified as F0-F1 (1) versus 15 patients classified as F2-F4 (2). *Mann–Whitney U-test p = 0.0024.

A significant correlation was found between SWE values and fibrosis (Rs = 0.465, p = 0.0006). Although SWE strongly correlated with fibrosis, there was some overlap between SWE values for consecutive Metavir stages. Therefore, SWE did not change significantly between F0 and F1 (p = 0.334), between F1 and F2 (p = 0.611), or between F3 and F4 (0.327), whereas a significant difference was observed after grouping F0-F2 and F3-F4 (p = 0.002) **(Table 2, Figs 1** and **2)**.

**Table 2:** Comparison of shear wave elastography values between different classes of Metavir scores.

	Shear Wave Elastography (kPa)		
	Median (range)	Median (range)	p*
**F0 vs F1**	4.60 (2.92–9.53)	5.37 (3.14–10.33)	0.3338
**F0 vs F2**	“	5.93 (4.53–6.91)	0.2936
**F0 vs F3**	“	7.94 (6.16–9.06)	0.0394
**F0 vs F4**	“	8.72 (7.35–16.66)	0.0042
**F1 vs F2**	5.37 (3.14–10.33)	5.93 (4.53–6.91)	0.6107
**F1 vs F3**	“	7.94 (6.16–9.06)	0.0426
**F1 vs F4**	“	8.72 (7.35–16.66)	0.0050
**F2 vs F3**	5.93 (4.53–6.91)	7.94 (6.16–9.06)	0.0330
**F2 vs F4**	“	8.72 (7.35–16.66)	0.0062
**F3 vs F4**	7.94 (6.16–9.06)	“	0.3272
**F0-F1 vs F2**	5.03 (2.92–10.33)	5.93 (4.53–6.91)	0.4084
**F0-F1 vs F3**	“	7.94 (6.16–9.06)	0.0305
**F0-F1 vs F4**	“	8.72 (7.35–16.66)	0.0025
**F0-F1 vs F2-F3-F4**	5.03 (2.92–10.33)	7.07 (4.53–16.66)	0.0024
**F0-F2 vs F3**	5.35 (2.92–10.33)	7.94 (6.16–9.06)	0.0238
**F0-F2 vs F4**	“	8.72 (7.35–16.66)	0.0017
**F0-F3 vs F4**	5.44 (2.90–10.33)	8.72 (7.35–16.66)	0.0026
**F0-F2 vs F3-F4**	5.35 (2.92–10.33)	8.72 (6.16–16.66)	0.0002

*Mann–Whitney U-test

Age, sex and Body mass index (BMI) were not significantly associated with SWE measurements.

**Fig 3:**
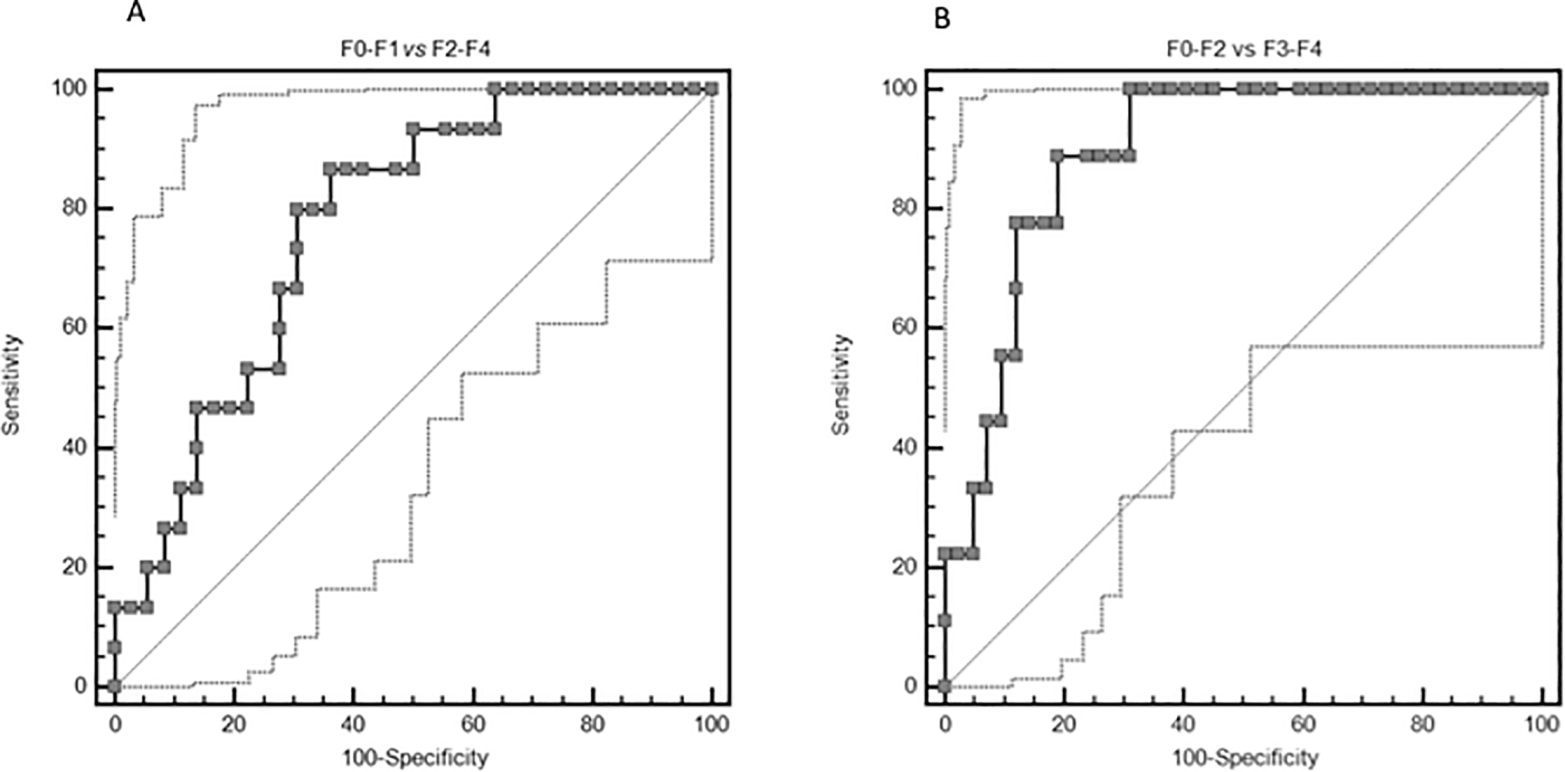
Receiver operating characteristic (ROC) curve for shear wave elastography at different fibrosis thresholds.

Using ROC curves (**Fig 3A** and **3B**), two threshold values for SWE were identified: 5.62 kPa for patients with fibrosis (≥F2; sensitivity 80%, specificity 69.4%, accuracy 77%) and 7.04 kPa for severe fibrosis (≥F3; sensitivity 88.9%, specificity 81%, accuracy 89%). **Table 3** summarizes the sensitivity, specificity, positive predictive value, and negative predictive value obtained using threshold values determined by Yuden’s statistic.

**Table 3:** ROC Curve shear wave elastography.

Parameter	F0-F1 vs F2-F4	F0-F1-F2 vs F3-F4
Cut-off (kPa)	5.62	7.04
Area under the curve	0.77 (0.63 to 0.88)	0.89 (0.78 to 0.96)
Sensitivity (%)	86.67 (59.50–98.30)	88.89 (51.80–99.70)
Specificity (%)	63.89 (46.20–79.20)	80.95 (65.90–91.40)
PPV (%)	50.00	50.00
NPV (%)	92.0	97.1

Diagnostic performance of Shear Wave Elastography for different cut-off values.

**Fig 4:**
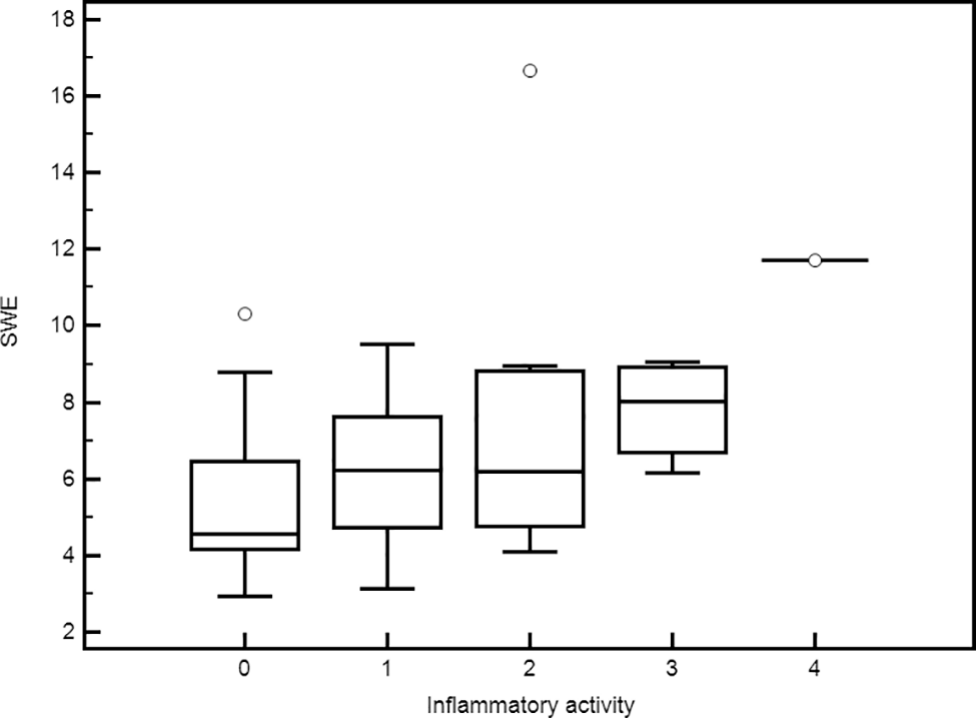
Box-and-whisker plots of shear wave elastography values for each Inflammatory activity stage. Liver stiffness values are reported on the y-axis, and inflammatory activity was reported on the x-axis. The line through each box represents the median, and the central box represents values from the lower to upper quartiles (25th- 75th percentile). Error bars show minimum and maximum non-extreme values.

SWE also correlated with inflammatory activity (Rs = 0.443, p = 0.0023) **(Fig 4)** and ALT levels (Rs = 0.287, p = 0.0804).

### Reproducibility

Interoperator reproducibility was assessed in 29 patients. The overall interobserver agreement analysed using the interclass correlation coefficient was 0.94 (CI 0.87–0.97); age >47 y was associated with increased interobserver ICC, whereas ICC was not influenced by BMI or gender **(Table 4)**. The ICC was poor for normal livers or mild fibrotic livers (F0-F1), whereas it was excellent for advanced fibrotic livers (F2-F4) **(Table 4)**.

**Table 4:** Influence of different parameters on interobserver agreement.

Variables	Intraclass Correlation Coefficient (95%)
**Sex**	
M	0.96 (0.88 to 0.98)
F	0.84 (0.43 to 0.95)
**Age**	
≤47	0.62(-0.32 to 0.89)
>47	0.95 (0.87 to 0.98)
**Body mass index**	
≤25	0.89 (0.62 to 0.97)
>25	0.95 (0.88 t0 0.98)
**Fibrosis Biopsy**	
(F0-F1)	0.39 (-1.27 to 0.83)
(F2-F3-F4)	0.95 (0.78 to 0.99)
Overall	0.94 (0.87 to 0.97)

**Fig 5:**
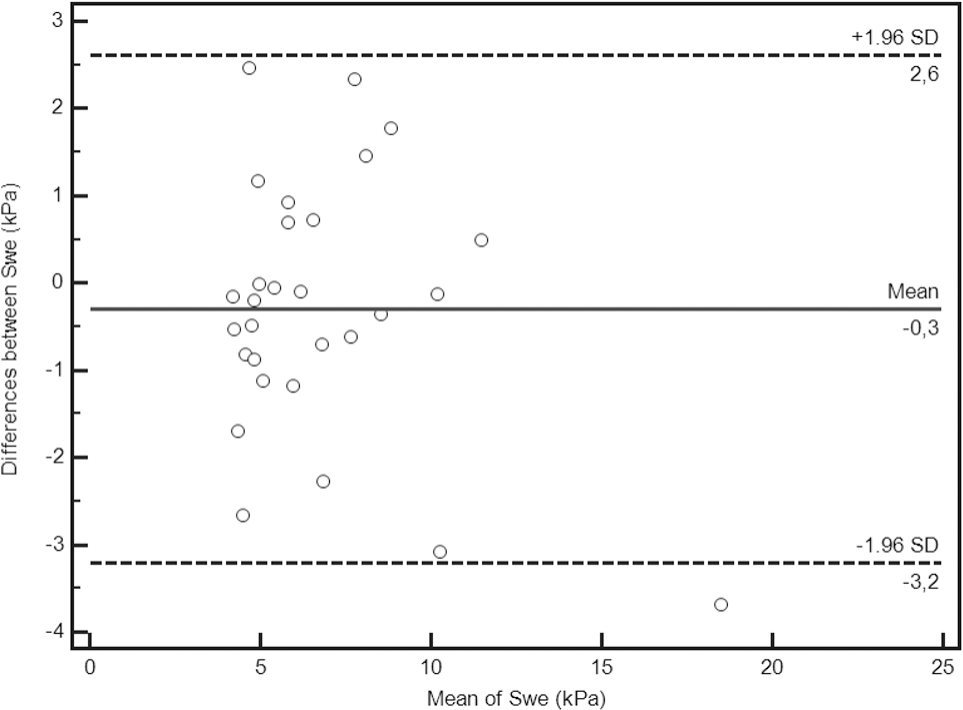
Bland-Altman analysis of the reproducibility of measurements. Bias is represented by the solid line (–0.3). The two dotted lines represent the limits of agreement for reproducibility (–3.2; 2.6).

The Bland-Altman plot **(Fig 5)** showed no systematic overestimation or underestimation between the two operators (mean difference -0.29 kPa and the 95% limits of agreement were -3.2 to 2.6).

Although SWE measurements were performed by two expert operators, some differences are expected because measurements performed on different days might be influenced by abdomen states such as meteorism that affect the ability to obtain liver images free from artefacts.

## Discussion

Currently, liver biopsy is considered the reference standard for CLD evaluation. However, biopsy is an invasive procedure, and histological examination by pathologists is time consuming, expensive and subject to interobserver variability [26, 27]. In addition, repeated liver sampling is unacceptable for clinical management and therefore cannot be used to determine the natural history of disease or the effect of therapies directed either at the fibrotic process itself or the underlying cause of CLD (such as antiviral treatment). In recent years, non-invasive methods to evaluate liver stiffness have been developed, and SWE could play an important role in the assessment of liver stiffness in clinical practice [28, 29, 30].

We have investigated the accuracy of SWE for liver fibrosis staging and assessed the interoperator reproducibility.

In our study, the median value and range were 4.6 kPa and 2.92–9.53 kPa, respectively, in normal liver and 6.15 kPa, and 3.14–16.7 kPa, respectively, in CLD. Moreover, measurements performed by different experienced operators showed high agreement and were not influenced by gender, age or BMI.

Our study partially confirms results from previously published studies.

Chong Hyun Suh et al. [31] evaluated normal values in 196 subjects with potential donors for living-donor liver transplantation using a biopsy-verified normal liver value (F0) and identified a cut-off of 6.2 kPa, which is close to our value of 5.6 kPa resulting from the ROC curve analysis. The authors also confirmed no effects on SWE from various confounding factors.

Ferraioli et al. [32] reported a cut-off of 7.1 kPa in 121 patients with chronic hepatitis C with F0-F1 Metavir staging. This value is higher than both our results and those of Chong et al, which might arise because of the different characteristics of examined subjects. In particular, SWE values might be higher in hepatitis C patients even without fibrosis compared to the values in normal subjects.

Sande J A et al. [33] conducted a multicentre prospective study on 128 patients with CLD to assess the accuracy of SWE alone and in combination with the aminotransferase platelet ratio index (APRI) score to predict biopsy results. The SWE median differentiated the Metavir subgroups F0-F1 and F2-F4 with an AUROC of 0.908 compared to 0.780 for the APRI score. When SWE and APRI were simultaneously utilized, an increase in the AUROC attributed to the APRI score was less than 1.2% higher than that predicted by the SWE median.

The SWE cannot distinguish intermediate fibrosis stages (stage F2 vs. F0-F1 **Table 2).** This might be clinically relevant, because according to the American Association for the study of liver disease, patients with hepatitis C genotype 1 infection should only be treated when moderate-severe fibrosis is observed [7, 34]. Therefore, monitoring with SWE would exclude only patients with severe fibrosis (F3-F4) from biopsy with an accuracy of 89.4%. Based on our findings, a biopsy should be performed on CLD patients with an SWE less than 7.04 kPa to identify those with F2 fibrosis.

In one subject with ascites, the liver stiffness measurement failed. Ascites are a physical limitation of the SWE measurement because the elastic waves propagate through liquids, attenuating their velocity. However, the presence of ascites indicates liver cirrhosis; therefore, the SWE evaluation is not necessary.

The overall ICC was 0.94; this is excellent considering that we compared measurements collected on different days. The ICC analysis in subgroups shows the most consistent results in patients with an advanced fibrotic state (F2-F4). The low variability in the measurements is certainly also due to the long experience of the operators (>20 years) in performing US examinations. The variability of measurements when the examinations are performed by operators with minimal experience must also be investigated.

Good agreement between operators is also the result of a methodology using the B-mode as a guide to place the box for SWE acquisition.

The results of this study show that SWE performed by skilled operators can be performed in almost all patients with CLD. SWE is reproducible; is not influenced by age, sex or BMI; and identifies patients with severe fibrosis. The introduction of SWE to clinical practice could immensely benefit patients and could greatly reduce the need for biopsies in patients with SWE lower than 5.6 kPa (F0-F1) or greater than 7.0 kPa (F3-F4). Moreover, the use of the SWE might meet the objective of identifying patients with no or mild fibrosis and patients with severe fibrosis; these patients are those that require more careful clinical and instrumental monitoring and have a priority indication for antiviral therapy.

Other studies [35–38] suggested that the extent of the necroinflammatory activity influences hepatic stiffness. Therefore, we also investigate the contribution of increasing of hepatic inflammation showing that in the setting of inflammation, defined by ALT, liver stiffness increased.

The main weakness of our work is that our data set is heavily weighted toward mild fibrosis therefore the results cannot be extended to entire population of patient with Chronic hepatic disease. Therefore the only cut-off that could be used in clinical practice is what separates normal subjects (F0-F1) from patients with fibrosis (>F1) that is 5.62 kPa. Moreover, the effects of other confounding factors (i.e., steatosis, iron deposition) were not evaluated.

Our findings suggest that transient elastography is a useful noninvasive method for assessment of fibrosis in chronic hepatitis patients. We have demonstrated the relationship between liver stiffness, fibrosis, and inflammatory activity.

Further studies are required to assess the predictive value for differentiating intermediate stages of fibrosis.
